# Beer as a Tonic

**Published:** 1890-09

**Authors:** 


### BEER AS A TONIC.

J. H. Kellogg, M. D., of Battle Creek, Mich., recently treated this subject at considerable length, in an able lecture, of which the following is a comprehensive extract.

The question is often asked, Is beer beneficial for persons weak from old age or other causes, and for those troubled with insomnia, as often recommended by physicians ? We know that the use of beer is often recommended in such cases by members of the medical fraternity ; and we have heard of people who take beer to make them sleep, and beer to keep them awake, take it in the winter as a protection against the cold, and in hot weather, to avoid feeling the heat. Alcohol is recommended as a general panacea for everything ; whereas, if we study carefully the principle upon which it acts, we shall find that it does nothing that is claimed for it. Alcohol claims to be a good stimulant, but it really makes people weak ; it claims to build a person up, when it really undermines his constitution. It is recommended to put people to sleep, but it does not remove the cause of sleeplessness ; it only acts as an anodyne.

It is a fallacy very commonly held that alcoholic liquors are excellent for old people, although it is admitted that they are bad for the young. The same argument might be used, and often is used, practically, in favor of the tobacco habit. Nearly every one says that tobacco is very bad for boys, and there is hardly a tobacco user so depraved that he will teach his own boy to smoke; yet middle aged and old men think they need it, or at least that it does them no harm.

Now, what change takes place between youth and old age which makes a thing which is harmful and poisonous in youth—for alcohol is a poison—beneficial and strengthening in old age? In old age there is a natural lessening of the bodily vigor, and a lowering of the vital powers. Fatty degeneration of the tissues begins to creep on. By means of it the walls of the blood vessels are weakened, and especially there is a fatty deposit in the small blood vessels of the brain, which robs them of elasticity as well as of strength. A sudden rush of blood to the head from any cause—excitement, passion or stimulation—may prove immediately fatal, or at least hasten dissolution.

The physiological effects of alcohol in any form, are to quicken the action of the heart, flush the face, and overcharge the brain with blood. The danger of apoplexy then is very great, to say nothing of other serious consequences. Alcohol accelerates the degeneration of tissue which is incident to old age; consequently, an aged person needs specially to abstain from stimulants; he needs to be more careful than a young person to avoid any thing which taxes or overloads his system. The advocates of alcohol for an old person, say that the bodily machinery is slowed down too much, and needs quickening. Nature has purposely put on the brakes, because there is always danger in high pressure upon an old machine. Certainly no engineer would take a nearly worn out engine to run a lightning express train. Nature puts the brakes on the human machine when it becomes enfeebled through the taking away of some of the natural energy, by making the muscles so weak that there shall be less temptation to work hard, or to run, or to do any violent thing which would quickly bring on heart failure. Then is it wise to take off the brakes which nature has put on? That is exactly what alcoholic stimulation does. It paralyzes the nerve centres of the brain, which control and regulate the blood vessels, and they relax, and the heart runs away at too rapid a rate. It is like a clock from which the pendulum weight has been taken; it will soon run down.

Then what alcohol really does for an old person is to hasten the day of his death, driving the human machinery at a rate incompatible with safety. His resistive powers are already low, and he needs to conserve his forces by well regulated, peaceful habits of life. His food and drink should be of the simplest kind, and he should avoid all manner of excitement, and all overtaxing of the mind or the body.

